# Pet owner perspectives, motivators and concerns about veterinary biobanking

**DOI:** 10.3389/fvets.2024.1359546

**Published:** 2024-02-20

**Authors:** Richard McEnhill, Holly Borghese, Sarah A. Moore

**Affiliations:** ^1^Blue Buffalo Veterinary Clinical Trials Office, Department of Veterinary Clinical Sciences, The Ohio State University, Columbus, OH, United States; ^2^MedVet Medical and Cancer Centers for Pets, Columbus, OH, United States; ^3^BluePearl Science, Tampa, FL, United States

**Keywords:** biospecimen, disease model, veterinary clinical trials, clinical research, biobank, animal

## Abstract

**Introduction:**

Veterinary biobanks store samples for future use and distribute samples to academic researchers and industry entities; however, informed consent provided by owners for pets contributing to biobanks can be complicated by limited understanding of goals, purpose, and logistics of biobanking.

**Methods:**

This survey-based study aimed to gather feedback from pet owners on how they viewed allowing their pet to contribute to a veterinary biobank, with the goal of identifying opportunities to improve education, awareness of veterinary biobanking initiatives, and the consent processes. An electronic survey was distributed to a listserv of 2,119 pet owners and responses were received from 118 respondents (5.6%).

**Results:**

Most respondents (67%) were not familiar with the concept of veterinary biobanking prior to having responded to the survey. Most (89%) were willing to allow their healthy pet to contribute samples to a veterinary biobanking program. Ninety-five percent would allow their sick pet to contribute. Most were neutral about financial incentives as a motivator to participate, although 40% indicated that if their pet’s condition resulted in a decision to humanely euthanize, they would be more likely to contribute to the biobank if the veterinary biobanking program covered the cost of euthanasia. Common concerns included security/confidentiality (36%), that results would not be shared with them (33%) or that samples would be used for other purposes beyond those advertised (22%).

**Discussion:**

These results suggest veterinary biobanking initiatives are well received by owners and most are willing to allow their pets to participate. Respondent concerns represent opportunities for veterinary biobanks to improve messaging and dissemination of results from work they support.

## 1 Introduction

Banking of tissues and biofluids from both healthy pets, and veterinary patients with various diseases has become a recent focus due to the ability of these samples to contribute to veterinary research and development as well as for their potential as a resource for translational research. Veterinary biobanks collect and store samples, with consent from pet owners, for future scientific use and often distribute samples to academic researchers and industry entities; however, informed consent provided by owners of pets contributing to biobanks can be hampered by limited understanding of overall goals, purpose, and logistics of biobanking. While interest in banked veterinary samples is growing, little is published with respect to owner perspectives and priorities related to veterinary biobanking (1–3).

Recent studies of human biobank contributors have demonstrated that most participants donate samples for altruistic reasons; however, lack of clarity can exist regarding potential for personal health benefits and governance of sample usage (4–6). These misconceptions might be further compounded by low health literacy in the general population (7). Similar factors have not been fully assessed in the context of veterinary biobanking and understanding pet owner priorities, concerns, and motivators for allowing biobank contributions can inform veterinary biobanking strategies but it is possible that, similar to what has been demonstrated on the human side, specific reservations or concerns might exist that could influence an owner’s willingness to allow their pet to participate. Additionally, the authors occasionally encounter concerns from attending clinicians about how owners might perceive being approached about biobanking. The purpose of this survey-based study was to gather feedback from pet owners on how they view allowing their pet to contribute to a veterinary biobank, with the goal of identifying opportunities to improve education, awareness of veterinary biobanking initiatives, and the consent processes.

## 2 Methods

### 2.1 Questionnaire

An electronic web-based survey (Qualtrics XM; Provo, UT) consisting of 51 questions was designed to collect information regarding demographics of respondents and their perspectives on an assortment of topics related to veterinary biobanking (Supplementary material S1). Questions included in the survey were adapted from similar studies evaluating patient perspectives on biobanking in human disease-specific research (8–10). After development, the survey was reviewed by The Ohio State University Institutional Review Board (IRB) and was determined to be exempt from the requirement for IRB approval (2021E0845). Consent for participation was requested from all participants before beginning the survey. After consenting to participate, respondents were asked to view an introductory video providing basic background information about veterinary biobanking to ensure that all respondents had cursory familiarity with the topic (11). This video was previously prepared by the Clinical and Translational Sciences Awards (CTSA) One Health Alliance veterinary biobanking subcommittee for the purposes of educating pet owners about veterinary biobanking.

After viewing the video, respondents answered a series of questions about their previous experience with both human and veterinary clinical trials, their pet’s overall health, their level of familiarity with veterinary biobanking, their perspective on allowing their pet to contribute to a veterinary biobank, and what they viewed as motivators for and concerns about participation. Questions were designed as check box or true/false formats; some questions allowed a selection of “other” where the respondent was provided a text box to expand briefly on their answer. The last question of the survey solicited other thoughts or concerns the respondent had related to veterinary biobanking that had not been covered in the survey and allowed free text entry.

### 2.2 Survey distribution

During the month of October 2021, a survey participation request was emailed to a list of individuals who had previously expressed interest in receiving communications from The Ohio State University Blue Buffalo Veterinary Clinical Trials Office. The survey included a consent form, demographic data collection, and video prompt, and several groups of questions assessing knowledge about and perspectives on veterinary biobanking programs. Informed consent was obtained from all respondents prior to participation. The invitation email provided an overview of the goals of the project, explained the process by which responses would remain anonymous, and indicated the intent to publish results of the survey in aggregate form. The survey was open for 2 weeks and a reminder email was sent 1 week after the initial invitation.

### 2.3 Statistical analysis

Results were summarized using descriptive statistics. Relationships between various demographic features and perspectives related to biobanking were assessed using Chi square analysis using the crosstabulation function in Qualtrics. Multiple comparisons were accounted for using a conservative Bonferroni method, and *p* values presented throughout the manuscript as corrected values are indicated with an asterisk. A *p* value <0.05 was considered significant for all analyses.

## 3 Results

### 3.1 Survey responses

**Figure 1:**
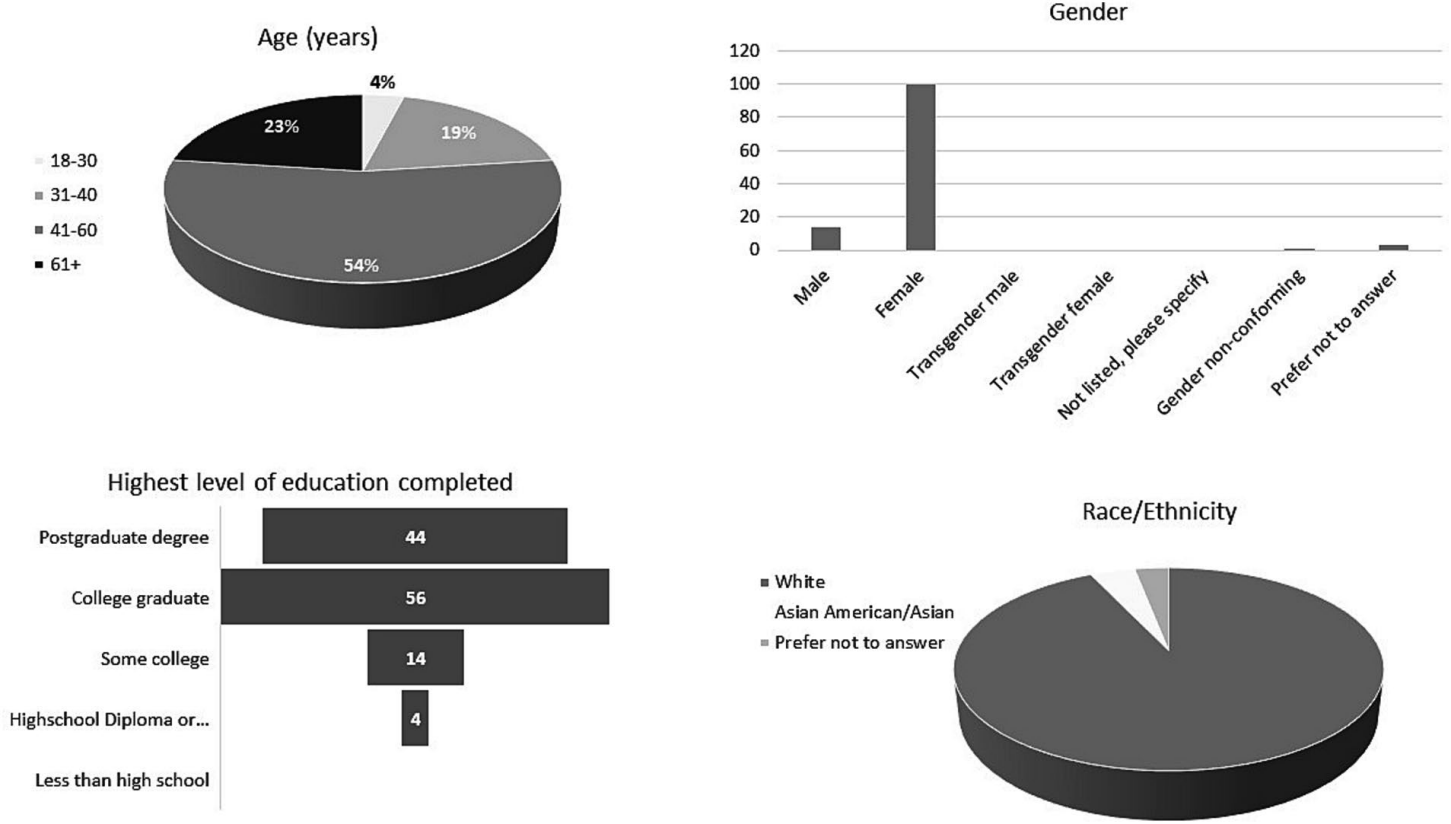
Demographics of 118 respondents to a web-based survey on veterinary biobanking.

The survey was distributed to 2,119 pet owners and 118 responses were received (5.6%). Respondent demographics are summarized in Figure 1. Most respondents were between 41 and 60 years of age (54%), were female (85%), reported their race/ethnicity as white (92%), listed their highest level of education as college graduate (56%) and had at least one pet residing in their household (99%). The most common pets owned by respondents of the survey were dogs (57%) and cats (29%).

### 3.2 Familiarity with veterinary biobanking

Of the 118 respondents, all but one indicated they had reviewed the introductory veterinary biobanking video prior to proceeding on in the study. Most (61%) indicated that neither they nor their pet had previously participated in a clinical trial and that their pet was not currently receiving treatment for a chronic or serious health condition (62%). For the 35% of respondents who indicated that either they or their pet had previously participated in a clinical trial, 25% of those had themselves been enrolled in a clinical trial and 74% had a pet who had participated in a veterinary clinical trial. The majority of respondents (67%) indicated that they were not familiar with the concept of veterinary biobanking prior to having responded to the survey.

### 3.3 Willingness to participate

When asked whether they would be willing to allow a healthy pet to contribute samples such as blood, urine, tissue or genetic materials to a veterinary biobanking program, most respondents (89%) indicated that they would, and only a few indicated that they would not (2%) or were unsure (8%). When the same question was asked in the context of a sick pet, defined as one being treated for a chronic or serious health condition, respondents were more willing to allow their pet to participate, with 95% responding yes, 1% responding no, and 4% indicating they were uncertain. Respondents were then asked about their willingness to allow collection and banking of different types of samples from their pet under various circumstances. Responses are summarized in Table 1. The strongest support was expressed for contributing waste samples such as left over blood or tissue when those samples were already being taken for medical purposes.

**Table 1:** Respondents to a survey on veterinary biobanking (*n* = 118) were asked to express their level of agreement with several statements related to willingness to allow their pet to contribute various sample types under different scenarios.

Sample type	Strongly agree	Somewhat agree	Neither agree nor disagree	Somewhat disagree	Strongly disagree
I would be willing to allow my pet to give an extra tube of blood or similar sample for research purposes if they were already having a tube of blood (or similar sample) taken for medical reason	85	11	2	0	2
I would be willing to allow my pet to have a tube of blood drawn for research purposes even though they did not need this procedure for medical reasons	44	31	6	16	3
I would be willing to give any samples, such as blood, that are left over from my pet’s medical tests for research purposes	92	5	2	0	1
If my pet were already having a piece of tissue or tumor removed for medical purposes, I would be willing to allow my pet to give a piece of that tissue for research purposes	94	3	2	0	1
I would allow my pet to contribute a biobanking program that was required to submit demographic information about myself and my pet to a central database (e.g., NIH or other national entity)	67	20	8	4	0
I would allow my pet to contribute to a biobanking program when repeated sampling was required (such as collection before and after surgical procedures or treatment)	53	31	12	3	1
Would you be willing to donate your pet’s DNA to a biospecimen repository? (phrased as a yes/no question)	Yes = 97			No = 3	

Table indicates % of respondents that indicated each level of agreement.

### 3.4 Knowledge of the research process, consent process, and sample ownership

Respondents were asked who they expected to benefit most from any samples their pet contributed to a veterinary biobank. Most indicated that they believed future veterinary patients would benefit most (70%), some indicated that veterinarians and scientists would benefit most (19%), a small group felt that their own pet would benefit most (7%) and a few respondents indicated “other” and entered free text answers with themes such as “both humans and animals,” “everyone” and “all of the above.”

Because for various types of health research, biological samples are often collected and stored for a very long time, respondents were asked who they thought owned the samples after their pet contributed them to the biobank. The institution where the research was being conducted was believed to be the owner by 67% of respondents, the funder of the research (if different from the institution) was believed to be the owner by 25% of respondents, and the researcher conducting the study was believed to be the owner by 7% of respondents.

The survey presented the respondent with a series of matrix true or false questions surrounding typical use cases and common scenarios involving samples once they have been contributed to a biobank. Responses are summarized in Table 2. Most respondents (94%) indicated understanding that researchers would not need to obtain their consent for use of the samples in the future and that results of future studies might not be reported to them (67%). Most (92%) also indicated understanding that their pet’s samples might be provided to researchers outside the institution where the samples were originally collected. A smaller number of respondents indicated that they believed that once their pet had contributed a sample, researchers would still need to obtain their consent to use that sample (6%); that they could choose how their pet’s samples were used in the future (10%); and that they would receive results from any studies resulting from their pet’s contribution (32%). Many respondents (42%) also believed they could request that their pet’s sample be removed from the bank at any time.

**Table 2:** Summary of responses from pet owners (*n* = 118) to statements related to understanding of the biobanking process.

Statement	True	False
Once my pet has contributed a sample, researchers must get my approval before using it	6	94
I will receive results from any studies that come from my pet’s contribution	32	68
I can choose how my pet’s samples are used	10	90
I can request that my pet’s sample be removed from the bank at any time	42	58
My pet’s sample will be linked to their medical record	57	43
My and my pet’s personal information will be kept confidential	92	8
Results from biobank studies will not be included in my pet’s medical record	64	36
My pet’s samples could be provided to researchers outside the institution where the samples were originally collected	92	8

Table indicates % of respondents that indicated each level of agreement.

### 3.5 Potential motivators of participation

Respondents were presented with a matrix of questions surrounding their motivation for allowing a pet to contribute samples to a veterinary biobanking initiative, where they were asked to rate their level of agreement with possible motivators from *Strongly agree* to *Strongly disagree*. Responses are summarized in Table 3. Respondents strongly agreed that they would allow their pet to participate because contributing would increase the chance of a cure for future animals with the same condition (91%); they hoped that their contribution would help another family and their pet with a similar condition (91%); because they wanted to help veterinarians with their efforts (89%); their pet, family or their other pets could benefit from the research (84%); or because they wanted to contribute to future research (80%). Most also strongly agreed that contributing would increase the chance of a cure for future humans with the same condition (73%) and many (40%) indicated they were neutral that financial incentives would make them more likely to allow their pet to participate. Many respondents also (40%) indicated that they would be more likely to contribute to a veterinary biobank at the time of their pet’s euthanasia if the veterinary biobanking program covered the cost of the euthanasia.

**Table 3:** Respondents to a survey on veterinary biobanking (*n* = 118) were asked to express their level of agreement with several statements related to potential motivators of participation in veterinary biobanking initiatives.

Statement	Strongly agree	Somewhat agree	Neither agree nor disagree	Somewhat disagree	Strongly disagree
I want to learn new information about my pet’s condition	69	18	8	3	3
My pet, my family, or our other pets could benefit from this research	84	12	3	1	0
Contributing will improve the number of treatment options available for my pet	64	19	13	3	0
Contributing will increase the chance of a cure for my pet	57	16	19	6	2
It’s important to help veterinarians with their efforts	89	11	0	0	0
I want to contribute to future research	81	16	3	1	0
I hope that my contribution helps another family and their pets with a similar medical condition	92	5	3	0	0
Contributing will increase the chance of a cure for future animals with the same condition	91	7	3	0	0
Contributing will increase the chance of a cure for future humans with the same condition	73	14	9	2	2
If my pet’s condition resulted in a decision to humanely euthanize them, I would be more likely to contribute to the biobank if the veterinary biobanking program covered the cost of the euthanasia	40	14	31	7	9
I would be more likely to consent to donation to the veterinary biobanking program if I received a financial incentive	18	14	40	9	19
I would not allow my pet to give a sample to a veterinary biobank	2	2	3	14	81

Table indicates % of respondents that indicated each level of agreement.

### 3.6 Concerns about participation

Respondents were presented with a matrix of questions surrounding potential concerns about allowing a pet to contribute samples to a veterinary biobanking initiative, where they were asked to rate their level of agreement with particular drivers of participation from *Strongly agree* to *Strongly disagree*. Responses are summarized in Table 4. Seventy-nine percent of respondents indicated that they either strongly or somewhat agreed with the statement “I do not have concerns about allowing my pet to contribute samples to a veterinary biobank.” Thirty-six percent strongly or somewhat agreed with concerns about security and confidentiality; and 33% strongly or somewhat agreed with concerns that results would not be shared with them.

**Table 4:** Respondents to a survey on veterinary biobanking (*n* = 118) were asked to express their level of agreement with several statements related to concerns about participation in veterinary biobanking initiatives.

Statement	Strongly agree	Somewhat agree	Neither agree nor disagree	Somewhat disagree	Strongly disagree
I am concerned about security and confidentiality	10	26	15	18	30
I am concerned that results will not be shared with me	8	25	26	14	25
I do not know enough about future uses or might not approve of them	3	8	21	26	41
I am concerned that my pet’s sample would be used for things other than the advertised purposes	5	17	19	26	32
I do not have concerns about allowing my pet to contribute samples to the veterinary biobank	47	32	11	8	3

Table indicates % of respondents that indicated each level of agreement.

### 3.7 Relationship between demographic factors and allowing pets to contribute

A significant relationship was not identified between most demographic factors and willingness to allow a healthy pet to contribute to veterinary biobanking initiatives (Table 5). Higher levels of education were associated with increased willingness to allow biobanking contributions from sick pets, where respondents who indicated a high school diploma or equivalent as their highest level of education had a higher frequency of a “no” response (*p* = <0.001*) than those with more advanced levels of education. Respondents who reported their race/ethnicity as “Asian American/Asian” had a higher response frequency of “no” about allowing their sick pet to contribute to biobanking (*p* = <0.001*), and those who indicated their religion was “atheist” or “agnostic” had a higher response frequency of “unsure” (*p* < 0.001*). A complete list of comparisons between demographic features and willingness to allow sick pets to participate can be found in Table 6.

**Table 5:** Associations between demographic factors and willingness to allow healthy pets to contribute samples such as blood, urine, tissue or genetic materials to a veterinary biobanking program.

Demographic factors	Willingness to allow a healthy pet to contribute to a biobank
Age	*p* = 0.698
Race/ethnicity	*p* = 0.142*
Gender	*p* = 0.369
Religious affiliation	*p* = 0.446*
Number of pets in the household	*p* = 0.662
Highest level of education completed	*p* = 0.087*
Previous familiarity with veterinary biobanking	*p* = 0.436
Has a pet with a chronic or serious health problem	*p* = 0.257
Previous clinical trial participation	*p* = 0.741

Response options were “yes,” “no” or “unsure”.

**Table 6:** Associations between demographic factors and willingness to allow a pet with chronic or serious health conditions (sick pets; b) contribute samples such as blood, urine, tissue or genetic materials to a veterinary biobanking program.

Demographic factors	Willingness to allow a sick pet to contribute to a biobank	Comments
Age	*p* = 0.815	
Race/ethnicity	*p* = 0.001*	Respondents identifying as Asian American/Asian responded “no” more frequently
Gender	*p* = 0.980	
Religious affiliation	*p* < 0.001*	Respondents identifying as Atheist or Agnostic responded “unsure” more frequently
Number of pets in the household	*p* = 0.848	
Highest level of education completed	*p* < 0.001*	Respondents with high school diploma or equivalent as highest level of education indicated “no” more frequently
Previous familiarity with veterinary biobanking	*p* = 0.917	
Has a pet with a chronic or serious health problem	*p* = 0.736	
Previous clinical trial participation	*p* = 0.622	

Response options were “yes,” “no” or “unsure”.

## 4 Discussion

This survey represents one of only a few published assessments of owners’ perspectives on veterinary biobanking. Cardy et al. recently evaluated perspectives on veterinary biobanking, predominately in pet owners residing in the UK, and specifically in the context of animal brain banking (2). Our results support those previously reported that most people are not inherently familiar with veterinary biobanking initiatives but after a cursory introduction, are highly receptive and supportive of the idea of allowing their pet to contribute. In the population surveyed in the present study, a large majority (89%) of pet owners were willing to allow their healthy pet to contribute to a veterinary biobank, and an even larger proportion (95%) were receptive to this in the context of a pet with a chronic or serious health condition. These results demonstrate the potential for biobanking educational programs to substantially increase participation in veterinary biobanking initiatives and suggest that clinicians might overestimate concerns about approaching owners to ask for consent to participate.

Respondents in the current study were predominantly middle-aged Caucasian women who owned dogs or cats. Geographic location of respondents was not assessed but presumably was primarily made up of pet owners residing in the Midwestern United States based on the listserv used for distribution. This likely reflects the make-up of our listserv and suggests that further work is needed to explore motivators and concerns across demographics. It also highlights an opportunity for expanded outreach to communities that are historically underserved with respect to veterinary health care in general, and certainly with respect to veterinary specialty referral care. As such, some bias clearly exists in the population surveyed in this study, as the survey was distributed via a non-random method to a convenience sample of owners who had self-selected future communications about clinical trials. The lack of diversity in our survey distribution pool limits interpretation of any associations identified between race/ethnicity and willingness to allow pets to contribute to biobanking initiatives, as non-Caucasian respondents represented only 8% of the those who answered the survey.

Our study also identified some areas of misconception or misunderstanding related to veterinary biobanking. These results are important because they can help to guide future educational initiatives that surround biobanking as well as highlight areas for improvement during the consenting process for biobank contributions. Motivators for contributing indicated by pet owners were mostly altruistic, with reasons such as the ability to help future pets, people and veterinarians being cited strongly by respondents as reasons to contribute.

Some differences in willingness to allow pets to participate in veterinary biobanking initiatives were noted based on respondent educational demographics, primarily where those with lower levels of education were less likely to indicate support for allowing their pet to contribute. This finding is consistent with studies evaluating impact of education on human clinical trial enrollment, where it is also noted that people without post-secondary education have a higher level of mistrust in clinical research and are less likely to express interest in enrollment and that higher health literacy scores are an independent predictor of both clinical research participation interest and eventual consent to enroll (12). Importantly, this impact can be mitigated by targeted educational intervention surrounding research participation (13). This highlights an important opportunity for future education around biobanking initiatives that could improve acceptability and participation across an important educational, and potentially socioeconomic, spectrum.

Due to our sampling approach, it is possible our cohort of respondents overestimates the acceptance of veterinary biobanking compared to the general population; however, it is unlikely the group overestimates concerns and barriers, of which several important points have been identified. First, 36% of respondents indicated that they had some level of concern about security and confidentiality. Enhanced focus on security and confidentiality, as well as educating owners about what data is catalogued along with their sample might help alleviate security concerns. Additionally, while most owners indicated understanding they might not receive results from studies of their pet’s samples, 33% also expressed concern about the fact that results would not be shared with them. This concern parallels one commonly expressed by contributors to human biobanks, and recent focus on scientific communication to patient stakeholders is an important approach to alleviating this concern, both in the context of individual results that might impact patient care, as well as broader impact from results of large-scale studies (14–16). Veterinary biobanks might consider enacting measures to disseminate research results to the general pet owning community, or to specific groups of biobank contributors.

In the current study, 22% of respondents indicated they had concerns that their pet’s samples could be used later for things other than originally advertised The issue of future use permissions is a timely topic in human biobanking as well, where some biobanks are shifting away from broad consent models allowing virtually unrestricted future use, based on the argument that broad consent for future unknown uses may not meet the definition of “informed consent” as it is articulated by the Common Rule and based on several legal cases that highlight the amount of misunderstanding and mistrust associated with broad consent models. Alternative consent models such as “categorical” or “tiered” consent models, where participants can opt in to allow their samples to be used for certain categories of research while restricting their use in other areas has been proposed although remains controversial (17, 18). A small but not insignificant number of respondents in the present study believed they could choose how their samples were used in the future, but our experience suggests that most veterinary biobanks use a broad consent model allowing relatively unrestricted use. The results of our survey might suggest revisiting this approach, or increasing education to ensure owners understand the meaning of broad use during the consenting process.

While altruistic motivators were common, 84% of owners also indicated strong agreement that potential benefit to their own pet, their own family, or their other pet’s health was a reason to contribute to veterinary biobanking. This is at odds with the overarching goal of most biobanks to bank samples for use in future studies, most of these likely to take place well after a tissue or biofluid donor has either been successfully treated for their own illness or passed away. This finding mirrors those from the human biobanking field, where patients commonly articulate perceived personal benefit from biobanking participation (19–22). This again underscores an opportunity to better highlight the goals of biobanking and emphasize that direct or indirect benefit to the patient themselves as being highly unlikely.

Incentivized participation is a controversial subject in the context of clinical trials, particularly with respect to what might cross the line to undue inducement. Conversely, incentives of various types, including financial, can encourage participation and are not uncommon in the human clinical trial and biobanking settings (23, 24). While no specific studies evaluate the influence of incentives on participation in veterinary biobanking, our data suggests that they might not be substantial drivers of participation, as most respondents were neutral on the role of financial incentives as a motivator for allowing their pet to participate; however, 40% of pet owners strongly agreed that if their pet’s condition resulted in a decision to humanely euthanize, they would be more likely to contribute to the biobank if the veterinary biobanking program covered the cost of the euthanasia. This concept might be an important consideration of veterinary biobanks as an important motivator for contribution in patients with chronic conditions who contribute longitudinal samples.

Overall, our results support that veterinary biobanking initiatives are generally well received by owners of both healthy and sick pets, and that most owners are willing to allow their pets to participate by contributing a variety of sample types when they are aware of the option to do so, underscoring the need for educational outreach to pet owners to help support enhanced biobanking participation. Respondents in the present study did express concerns around information security and whether results from studies using their pet’s samples would ever be shared with them. These represent two actionable areas where veterinary biobanks can work to improve their messaging around data security measures already in place, as well as outreach and dissemination of results from works supported by the biobank. Some misconceptions were also present among respondents, primarily related to ability to stipulate how their pet’s samples would be used and potential linkage to clinical information associated with their pet. These areas represent important opportunities for education during the consenting process and for consideration of process improvements that could allow for tiered or categorical consent for use of biospecimens.

## Data availability statement

The raw data supporting the conclusions of this article will be made available by the authors, without undue reservation.

## Author contributions

RM: Data curation, Investigation, Methodology, Writing – original draft, Writing – review & editing. HB: Conceptualization, Writing – original draft, Writing – review & editing, Data curation. SM: Conceptualization, Writing – original draft, Writing – review & editing, Formal analysis, Investigation, Methodology, Project administration, Supervision.
