# Supplementary material for: Pet owner perspectives, motivators and concerns about veterinary biobanking
Source: Front Vet Sci. 2024 Feb 20;11:1359546. doi: 10.3389/fvets.2024.1359546 (PMC10912476; doi:10.3389/fvets.2024.1359546)
Supplement: Supplementary file 1 [file Data_Sheet_1.PDF]

Thank you for your interest in our survey regarding pet owners' perspectives on

Thank you for your interest in our survey regarding pet owners' perspectives on veterinary biobanking. The goal of our survey is to gather feedback on how pet owners view allowing their pet to contribute samples such as blood or tissue to a biobank (also called a tissue bank or bio specimen repository). This survey will ask you to view a brief video providing some background on veterinary biobanking, and then to answer a few questions about yourself, your pet and your perspectives on biobanking. We anticipate it will take no more than 10 minutes to complete this survey.

This survey was evaluated by The Ohio State University Institutional Review Board (IRB) and granted an exemption. Participation in the survey is entirely voluntary and you may choose to exit the survey at any time prior to completion (at which point your responses will not be recorded). Your responses are anonymous, cannot be connected to you or your pet, and will not influence the healthcare your pet receives now or in the future. Results of the survey may be used for designing future educational initiatives related to veterinary biobanking, and may be published in whole or in part. If shared outside the research team, all responses will be presented in aggregate form only.

Based on this information, do you consent to participate in the survey regarding pet owner perspectives on veterinary biobanking?

- ○ Yes
- ○ No

Thank you for your interest. You are not eligible to complete the survey.

Owner Demographics

Please select your age category:

- ○ 18-30
- ○ 31-40
- ○ 41-60
- ○ 61+

To which gender do you most identify?

- ○ Male
- ○ Female
- ○ Transgender Male
- ○ Transgender Female
- ○ Gender non-conforming
- ○ ______ Not listed (Please specify)
- ○ Prefer not to answer

To which race/ethnicity do you most idenfity?

- ○ White
- ○ African American/Black
- ○ American Indian or Alaska Native
- ○ Asian American/Asian
- ○ Native Hawaiian or Pacific Islander

- ◯ [ ] Other (please specify)
- ◯ Prefer not to answer

To which religious affiliation do you most identify?

- ◯ Christian
- ◯ Jewish
- ◯ Muslim
- ◯ Hindu
- ◯ Buddhist
- ◯ Sikh
- ◯ Taoist
- ◯ Deist
- ◯ Atheist
- ◯ Agnostic
- ◯ [ ] Other (Please specify)
- ◯ Prefer not to answer

What is the highest level of education you have completed?

- ◯ Postgraduate Degree
- ◯ College Graduate
- ◯ Some College
- ◯ High School Diploma or Equivalent
- ◯ Less than High School

Number of pets currently residing in your household:

- ◯ 0
- ◯ 1

- 2
- 3
- 4
- 5+

Which species of pets do you currently have?

- Cats
- Dogs
- Horses
- Other

Video Prompt

Before completing the remainder of this survey, please take a few moments to review the video on veterinary biobanking, which can be accessed here:

Veterinary Biobanking Informational Video

This video is brief, and will take approximately 3 minutes to review.
Once you have reviewed the video, please proceed to the following questions.

Did you review the video on veterinary biobanking?

- Yes
- No

Preliminary for General Questions

Have you or your pet ever participated in a clinical trial?

- ◯ Yes
- ◯ No
- ◯ Unsure/Cannot Recall

Do you currently have a pet receiving treatment for a chronic or serious health condition?

- ◯ Yes
- ◯ No

General Questions - Yes #1

Please select any statements that are correct about clinical trial participation:

- ☐ I have been a participant in a human clinical trial
- ☐ My pet has participated in a veterinary clinical trial

General Questions - Yes #2

How would you rate your pet's current state of health?

- ◯ Excellent
- ◯ Good
- ◯ Fair
- ◯ Neutral
- ◯ Poor
- ◯ Very Poor

How likely do you feel your pet will be cured of their condition?

- ◯ Very likely to be cured (greater than 80%)
- ◯ Likely to be cured (60-80%)
- ◯ Possible to be cured (40-59%)
- ◯ Unlikely to be cured (20-39%)
- ◯ Very unlikely to be cured but still possible (less than 20%)
- ◯ No chance of cure

General Questions - #2

Before today, were you familiar with the concept of veterinary biobanking?

- ◯ Yes
- ◯ No
- ◯ Unsure/Cannot Recall

If your pet were healthy (not currently being treated for a chronic or serious health condition), would you consider consenting for your pet to contribute samples (blood, urine, tumor tissue, genetic material) to a veterinary biobanking program?

- ◯ Yes
- ◯ No
- ◯ Unsure

If your pet were sick (currently being treated for a chronic or serious health condition, would you consider consenting for your pet to contribute samples

(blood, urine, tumor tissue, genetic material) to a veterinary biobanking program?

- Yes
- No
- Unsure

If you consented for your pet to contribute samples to a veterinary biobank, who would you expect to benefit most?

Please select only one answer.

- My pet
- Future veterinary patients
- Veterinarians and scientists
- [ ] Other (please specify)

Rating Questions #1

Please rate your level of agreement with each of the following statements:

	Strongly Agree	Somewhat Agree	Neither Agree nor Disagree	Somewhat Disagree	Strongly Disagree
I would be willing to allow my pet to give an ***extra*** tube of blood or similar sample for research purposes if they were **already** having a tube of blood (or similar sample) taken for medical reasons	○	○	○	○	○

	Strongly Agree	Somewhat Agree	Neither Agree nor Disagree	Somewhat Disagree	Strongly Disagree
I would be willing to allow my pet to have a tube of blood drawn for research purposes even though they did not need this procedure for medical reasons	○	○	○	○	○
I would be willing to give any samples, such as blood, that are left over from my pet's medical tests for research purposes	○	○	○	○	○
If my pet were already having a piece of tissue or tumor removed for medical purposes, I would be willing to allow my pet to give a piece of that tissue for research purposes	○	○	○	○	○

Please rate your level of agreement with each of the following statements:

	Strongly Agree	Somewhat Agree	Neither Agree nor Disagree	Somewhat Disagree	Strongly Disagree
I would allow my pet to contribute a biobanking program that was required to submit demographic information about myself and my pet to a central	○	○	○	○	○

	Strongly Agree	Somewhat Agree	Neither Agree nor Disagree	Somewhat Disagree	Strongly Disagree
database (e.g. NIH or other national entity)					
I would allow my pet to contribute to a biobanking program when repeated sampling was required (such as collection before and after surgical procedures or treatment)	○	○	○	○	○
I would want to be informed of results of important studies that might come from samples my pet provided	○	○	○	○	○
If my pet's condition resulted in a decision to humanely euthanize them, I would be more likely to contribute to the biobank if the veterinary biobanking program covered the cost of the euthansia	○	○	○	○	○
I would be more likely to consent to donation to the veterinary biobanking program if I received a financial incentive	○	○	○	○	○

General Questions - #3

The genetic code for some diseases is embedded in an animal's DNA. DNA can be extracted from samples such as blood. Would you be willing to donate your pet's DNA to a biospecimen repository?

- Yes
- No

For various types of health research, biological samples (such as blood, tissue, urine, and genetic information) are often collected and stored long term. If your pet provided a sample that was part of such a collection, who do you think owns these samples?

Please select only one answer.

- You
- The searcher conducting the research study
- The institution where the research is being conducted
- The entity funding the research (if different from above)

True False Questions

Regarding allowing your pet to contribute to a veterinary biobank, please indicate if you believe the following statements are true or false:

	True	False
Once my pet has contributed a sample, researchers must get my	○	○

	True	False
approval before using it		
I will receive results from any studies that come from my pet's contribution	○	○
I can choose how my pet's samples are used	○	○
I can request that my pet's sample be removed from the bank at any time	○	○
My pet's sample will be linked to their medical record	○	○
My and my pet's personal information will be kept confidential	○	○
Results from biobank studies will not be included in my pet's medical record	○	○
My pet's samples could be provided to researchers outside the institution where the samples were originally collected	○	○

Rating Questions #3

Please rate your level of agreement with each of the following statements regarding **<u>why you might consent</u>** to allow your pet to give a sample to a veterinary biobank.

	Strongly Agree	Somewhat Agree	Neither Agree nor Disagree	Somewhat Disagree	Strongly Disagree
I want to learn new information about my pet's condition	○	○	○	○	○
My pet, my family, or our other pets could benefit from this research	○	○	○	○	○
Contributing will improve the number of treatment options available for my pet	○	○	○	○	○
Contributing will increase the chance of a cure for my pet	○	○	○	○	○
It's important to help veterinarians with their efforts	○	○	○	○	○
I want to contribute to future research	○	○	○	○	○
I hope that my contribution helps another family and their pets with a similar medical condition	○	○	○	○	○
Contributing will increase the chance of a cure for future animals with the same condition	○	○	○	○	○
Contributing will increase the chance of a cure for future humans with the same condition	○	○	○	○	○
I would not allow my pet to give a sample	○	○	○	○	○

	Strongly Agree	Somewhat Agree	Neither Agree nor Disagree	Somewhat Disagree	Strongly Disagree
to a veterinary biobank					

Rating Questions #4

Please rate your level of agreement with each of the following statements regarding **concerns you might have** about allowing your pet to contribute to the veterinary biobank:

	Strongly Agree	Somewhat Agree	Neither Agree nor Disagree	Somewhat Disagree	Strongly Disagree
I am concerned about security and confidentiality	○	○	○	○	○
I am concerned that results will not be shared with me	○	○	○	○	○
I don't know enough about future uses or might not approve of them	○	○	○	○	○
I am concerned that my pet's sample would be used for things other than the advertised purposes	○	○	○	○	○
I do not have concerns about allowing my pet to contribute samples to the veterinary biobank	○	○	○	○	○

What other comments or concerns related to veterinary biobanking would you like

Are there any additional comments or concerns related to veterinary biobanking that you would like to share?
